# Mitotic phosphorylation of Tau/MAPT modulates cell cycle progression in prostate cancer cells

**DOI:** 10.1007/s00432-023-04721-2

**Published:** 2023-03-31

**Authors:** Letizia Clementi, Samantha Sabetta, Veronica Zelli, Chiara Compagnoni, Alessandra Tessitore, Vincenzo Mattei, Adriano Angelucci

**Affiliations:** 1 Department of Biotechnological and Applied Clinical Sciences, University of L’Aquila, Via Vetoio, 67100 L’Aquila, Italy; 2 Center for Molecular Diagnostics and Advanced Therapies, University of L’Aquila, 67100 L’Aquila, Italy; 3 Biomedicine and Advanced Technologies Rieti Center “Sabina Universitas”, 02100 Rieti, Italy

**Keywords:** Prostate cancer, Docetaxel, Cancer therapy, Mitosis, Cell cycle

## Abstract

**Purpose:**

Tau/MAPT (microtubule associated protein tau) protein is actively studied for the pathologic consequences of its aberrant proteostasis in central nervous system leading to neurodegenerative diseases. Besides its ability to generate insoluble toxic oligomers, Tau homeostasis has attracted attention for its involvement in the formation of the mitotic spindle. This evidence, in association with the description of Tau expression in extra-neuronal tissues, and mainly in cancer tissues, constitutes the rationale for a more in-depth investigation of Tau role also in neoplastic diseases.

**Methods:**

In our study, we investigated the expression of phosphorylated Tau in prostate cancer cell lines with particular focus on the residue Thr231 present in microtubule binding domain.

**Results:**

The analysis of prostate cancer cells synchronized with nocodazole demonstrated that the expression of Tau protein phosphorylated at residue Thr231 is restricted to G2/M cell cycle phase. The phosphorylated form was unable to bind tubulin and it does not localize on mitotic spindle. As demonstrated by the use of specific inhibitors, the phosphorylation status of Tau is under the direct control of cdk5 and PP2A, while cdk1 activation was able to exert an indirect control. These mechanisms were also active in cells treated with docetaxel, where counteracting the expression of the dephosphorylated form, by kinase inhibition or protein silencing, determined resistance to drug toxicity.

**Conclusions:**

We hypothesize that phosphorylation status of Tau is a key marker for G2/M phase in prostate cancer cells and that the forced modulation of Tau phosphorylation can interfere with the capacity of cell to efficiently progress through G2/M phase.

## Introduction

Tau/MAPT, a phosphoprotein belonging to the family of microtubule associated proteins (MAPs), is mainly expressed in neuronal models where it is intensely studied for its key involvement in neurodegenerative diseases (Delacourte and Buée 2000). However, Tau expression was reported also in breast, colorectal, pancreas, prostate and stomach tissues and their cancer counterparts where its pathophysiology action is largely unexplored. Tau is a putative multifunctional protein with the main role derived from its ability to bind microtubules by a microtubule-binding domain in the C-terminal region. The human Tau protein is encoded by the MAPT (Microtubule Associated Protein Tau) gene consisting of 16 exons and located on the long arm of chromosome 17. Alternative splicing of the three main exons (2, 3 and 10) results in the expression in brain of six Tau isoforms ranging from 352 to 441 in length, with a molecular weight between 50 and 70 kDa (Andreadis 2005).

In addition to the differential processing of the primary transcript, the Tau protein is the target of complex post-translational modifications that include phosphorylation of the microtubule binding domain particularly at residues Ser262, Thr231 and Ser235 (Alquezar et al. 2020). Site-specific phosphorylation is regulated by a balanced activity between different protein kinases and phosphatases, which thus play an important role in controlling the affinity of Tau protein for microtubules (Stoothoff and Johnson 2005). Mutation-independent molecular events promoting hyper-phosphorylation of the Tau protein are associated with the acquisition of pathological functions. Indeed, hyper-phosphorylated Tau loses its ability to bind tubulin and begins a process of self-aggregation into progressively larger, insoluble structures called oligomers that compromise cellular survival (Ward et al. 2012). Intracellular Tau accumulation has long been associated with neurodegenerative diseases, including Alzheimer's disease (AD), Parkinson's disease, progressive supranuclear palsy, corticobasal degeneration and frontotemporal dementias, collectively known as tauopathies. At the same time, early evidence also suggests that aberrant post-translational Tau modifications are involved in cell cycle defects in tauopathies resulting from defective cell cycle re-entry (Preuss and Mandelkow 1998; Andorfer et al. 2005). A study in Drosophila model demonstrated that the deleterious effects of human Tau are dependent on its phosphorylation status and tubulin-binding ability and occur also in the absence of Tau filament/neurofibrillary tangle formation (Cowan et al. 2010). In addition, Tau overexpression was a sufficient condition in Drosophila for inducing a mitotic block leading to aneuploidy (Bougé and Parmentier 2016). Significantly, similar results have been replicated in neuroblastoma SH-SY5Y cell line, forcing the expression of WT Tau, with the formation of aberrant mitotic spindle (Malmanche et al. 2017).

The role of Tau proteostasis in controlling mitosis execution, as suggested by the studies of tauopathies, opened a speculative window for evaluating its contribute also to cancer progression. It is worth of note that Tau protein is frequently neo-expressed in tumor cells, in a variable percentage of patients with different tumors, including prostate cancer (PCa) (Schroeder et al. 2019). The expression of Tau protein, analyzed by immunohistochemistry in PCa, was restricted to a subset of patients at worse prognosis and was correlated with androgen therapy resistance (Sekino et al. 2020). PCa cell lines express high level of multiple Tau splice variants, and a phosphorylated pattern similar to that observed in tauopathies (Souter and Lee 2009). Previous studies in breast cancer have shown that tumor expression of Tau can alter sensitivity to taxanes with significant implication in patients survival (Ikeda et al. 2010; Shao et al. 2010). Involvement of Tau in taxanes resistance was suggested by us and other authors also in PCa cell lines (Yang et al. 2017; Martellucci et al. 2021). In our previous work, we hypothesized that in PCa cell lines accumulation of Tau oligomers was associated with aberrant mitotic spindle and sensitizes to docetaxel cytotoxicity.

In this study, we aimed at investigating molecular mechanisms involved in the phosphorylation of Tau protein in PCa cell lines, and their role in G2/M progression. In addition, we verified whether these mechanisms were relevant in explaining sensitivity to docetaxel treatment.

## Materials and methods

### Cell lines

Experiments were performed using PC3 cell line (derived from bone metastases of human prostate cancer), DU145 cell line (derived from central nervous system metastases of human prostate adenocarcinoma) and LNCaP cell line (androgen-dependent prostate cancer). The PC3 cells were cultured in DMEM high glucose growth medium and DU145 and LNCaP cells in RPMI 1640 medium. For all cell lines the growth medium was supplemented with 10% fetal bovine serum, 2 mM glutamine 100 IU/ml penicillin and 100 μg/ml streptomycin. Cell lines were supplied by ECACC and underwent regularly testing for mycoplasma by Hoechst DNA staining and PCR. Authentication procedures included species verification by DNA barcoding and identity verification by DNA profiling. Human cell lines were analyzed by PCR of short tandem repeat sequences within chromosomal microsatellite DNA (STR-PCR). DU145, LNCaP and PC3 cells were initially plated at a density of 10^4^ cells/cm^2^, and incubated in 5% CO_2_ at 37 °C. Cell number and viability was analyzed using a Neubauer hemocytometer chamber and by the trypan blue dye (Sigma-Aldrich, St. Louis, Missouri, USA) exclusion test. Cells synchronization was achieved using the microtubule polymerization inhibitor nocodazole (Enzo Life Sciences, Farmingdale, NY, USA). Briefly, cells were seeded at a density of 50,000 cells/cm^2^ and after 24 h, were treated with 60 ng of Nocodazole for 18 h. Washout was performed removing the culture medium and gentle washing twice cells with phosphate buffered saline (PBS). Roscovitine (Cell Signaling Technology, Danvers, MA, USA) is a selective inhibitor of cyclin-dependent cdk5 kinase and was used at a concentration of 10 μM. RO-3306 (Enzo Life Sciences USA) is a cdk1 inhibitor and it was used at a concentration of 10 μM. Okadaic acid (Cell Signaling Technology, Danvers, MA, USA) is a serine/threonine protein phosphatase PP2A inhibitor and it was used at a concentration of 20 nM.

### Western blot

Cancer cells were processed for protein extraction with cell lysis buffer containing 0.1% Triton X-100, 10 mM Tris–HCl (pH 7.5), 150 mM NaCl, 5 mM EDTA, and supplemented with 1 mM Na_3_VO_4_ and 75 U of aprotinin (Sigma-Aldrich), and incubated for 20 min at 4 °C. The cell lysate was centrifuged for 10 min at 1300*g* to eliminate nuclei and large cellular debris. After protein concentration analysis by Bradford Dye Reagent assay (Bio-Rad, Hercules, CA, USA), the whole cell lysate of each sample was subjected to 10% sodium-dodecyl sulphate polyacrylamide gel electrophoresis (SDS-PAGE) together with prestained protein molecular markers SHARPMASS VII (Euroclone, Milan, Italy). The proteins were electrophoretically transferred onto nitrocellulose membranes Amersham protran 0.2 μm (Cytiva Europe, Freiburg, Germany) for 90 min at 350 mA. Membranes were blocked for 1 h at RT with 10% nonfat milk in Tris Buffered Saline (Bio-Rad) at pH 7.4 containing 20 mM Tris, 500 mM NaCl and supplemented with 0.05% Tween 20 (Bio-Rad), and probed for 1 h at RT with primary antibodies according to dilution suggested by manufacturer: anti-Tau (D1M9X), anti-Phospho-Tau (Thr231) anti-pH2A.X (D7T2V), anti-H2A.X, Cyclin B1 (D5C10) and anti-GAPDH (D16H11) (all from Cell Signaling Technology, Danvers, MA, USA). Protein bands were visualized after 1 h of incubation with horseradish peroxidase (HRP)-conjugated anti-rabbit IgG or anti-mouse IgG (Cell Signaling Technology, Danvers, MA, USA) at RT, and then with chemiluminescence reagents (Amersham, Buckinghamshire, UK). Chemiluminescent signals were acquired by Chemidoc XRS system and digitally analyzed for determination of band molecular weight and density by Imagelab software (Bio-Rad).

### Knockdown of Tau protein by siRNA

Cells were seeded at a density of 5 × 10^4^ cells/mL in a 6 well-plate and in standard culture conditions. Twenty-four hours after seeding, a pool of four different siRNA constructs (Qiagen, Valencia, CA, USA) was diluted in 200 µl culture medium without serum to obtain a final concentration of 20 nM. 10 µl of HiPerFect Transfection Reagent (Qiagen) were added to the diluted siRNA, vortexing. Samples were incubated for 5–10 min at room temperature then added drop-wise onto the cells. The cells were incubated with the transfection complexes under their normal growth conditions and gene silencing was checked after 48 h by western blot. As negative control, cells were transfected with 20 nM scrambled siRNA (AllStars Negative Control, Qiagen).

### Immunofluorescence

Cells were seeded at a density of 10,000 cells/cm^2^ on glass coverslips pretreated with 30 μg/mL poly-L lysine to promote adherence. At the end of the treatment cells were fixed with 4% paraformaldehyde (Euroclone) for 10 min at 4 °C and permeabilized for 10 min at RT with 0.1% (v/v) Triton X-100 (Bio-Rad). After washing, cells were incubated, according to dilution suggested by manufacturer, with anti-Tau (D1M9X) anti-Phospho-Tau (Thr231) or anti-β-Tubulin (D3U1W) (all from Cell Signaling Technology), for additional 45 min. After washing with PBS, cells were incubated for 30 min at RT with Alexa Fluor 594/488-conjugated secondary antibodies (Jackson ImmunoResearch Laboratories, West Grove, PA, USA). Controls were performed by omitting the primary antibody. Slides were mounted with ProLong Gold antifade mounting medium with DAPI (Cell Signaling Technology). Finally, cells were observed with Zeiss Axio Vert. A1 fluorescence microscope (Zeiss, Jena, Germany) and acquired images were digitally elaborated with a modular image-processing and analysis software (Zen 2012 SP2 Blue Edition).

### Flow cytometry analysis

Cell cycle analysis by cytometer was performed following standard staining protocol. Briefly, at the endpoint cells were washed twice in PBS and fixed in 70% ethanol for 10 min at 4 °C. Then, cells were washed twice with PBS, resuspended in 0.5 mL PBS, 50 μL RNase A (5 μg/mL) (Sigma-Aldrich), and stained with 0.5 mL of 100 mg/mL propidium iodide (Sigma-Aldrich) in PBS. Cells were incubated for 30 min at room temperature in the dark and analyzed for DNA content and the fluorescence intensity was measured using the Accuri C6 cytometer (BD Technologies, Durham, NC, USA).

### Immunoprecipitation assay

Anti β-tubulin antibody (D20G3) (Cell Signalling Technology) was incubated for 2 h at 4 °C with protein-G Agarose Beads (Cell Signalling Technology) and Protein A-Agarose Beads (Santa Cruz Biotechnology) in Tris 50 mM pH 7.4, NP40 1%, Na-deoxycholate 0.25%, NaCl 150 mM and EDTA 1 mM (buffer 1). After centrifugation for 1 min at 3000 rpm the agarose beads bound to antibody (anti-tubulin-beads) were washed five times in Tris 50 mM pH 7.4, NP40 1%, Na-deoxycholate 0.25% and EDTA 1 Mm (buffer 2). Subsequently, cell lysis of the samples was performed on ice for 30 min with buffer 1 supplemented with protease and phosphatase inhibitors cocktail (Sigma-Aldrich). After centrifugation at 12,000 rpm for 10 min, the resulting cell lysate was incubated at 4 °C overnight on a rotary device with anti-tubulin-beads in buffer 1. After centrifugation for 1 min at 6000 rpm, beads were washes five times with buffer 2, while the first supernatant representing the immunodepleted sample was stored at -20 °C. Finally, immunoprecipitated samples were boiled for 3 min, and centrifuged at 13,400 rpm for 1 min. The immunoprecipitated and immunodepleted proteins were resolved by SDS-PAGE and analyzed by Western blotting.

### Publicly available datasets

Gene Expression Omnibus (GEO) GSE33455, GSE158494 and GSE36135 datasets were used to evaluate the expression levels of MAPT in DU145, PC3 and 22Rv1 prostate cancer cell lines (DU145 and PC3 in GSE33455 and GSE158494 datasets, DU145 and 22Rv1 in GSE36135 dataset) sensitive and resistant to docetaxel.

### Data analysis and statistics

All the statistical procedures were performed by GraphPad Prism Software Inc. (San Diego, CA, USA). Data are expressed as means ± standard deviations (SD) of at least three independent experiments. The statistical significance between measure series was calculated with parametric Student t test and p values of less than 0.05. Gene expression data were analyzed using GEO2R software (R 3.2.3, Biobase 2.30.0, GEOquery 2.40.0, limma 3.26.8); an adjusted *p* value < 0.05 (Benjamini–Hochberg FDR correction for multiple testing) was considered statistically significant.

## Results

### Tau is phosphorylated at residue Thr231 during G2/M phase of cell cycle

**Fig. 1:**
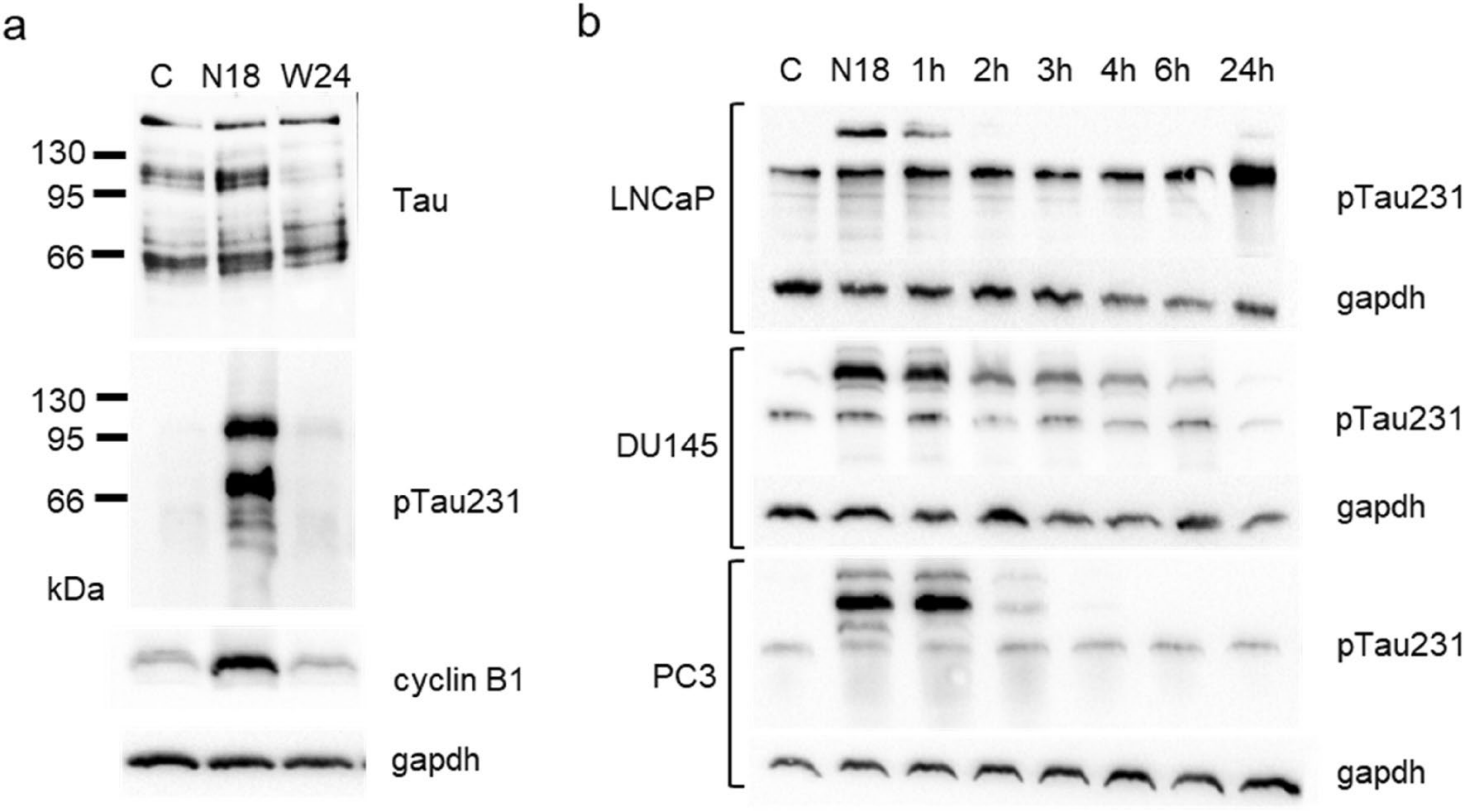
Tau protein electrophoretic pattern visualized by western blot after cell cycle synchronization induced by nocodazole. **a** Total cell lysates from control DU145 cells (C), cells treated for 18 h with nocodazole (N18) and cells treated for 18 h with nocodazole and then washed out for further 24 h (W24), were analysed for the expression of total Tau, Tau phosphorylated at Thr231 (pTau231) and cyclin B1. **b** Total cell lysates from LNCaP, DU145 and PC3 cells were subjected to western blot analysis for the expression of Tau phosphorylated at Thr231 (pTau231). All cell lines were treated for 18 h with nocodazole and cell lysates were recovered at 18 h (N18) and in a time course after washing out (1 h, 2 h, 3 h, 4 h, 6 h, 24 h). For each western blot gapdh expression was showed as loading control

Tau protein when analysed by western blot generated a multiband pattern in which we can individuate different monomeric forms span from approximately 50 kDa to 70 kDa and oligomers detected as bands higher than 100 kDa (Fig. 1a top panel). The incubation for 18 h of DU145 cells with nocodazole, a well-known antimitotic agent interfering with polymerization of microtubules, determined an accumulation of oligomers around 100 kDa in parallel with the expected increase in cyclin B1 (Fig. 1a). Because generation of soluble Tau oligomers is dependent on its phosphorylation status, we analysed the expression of the Tau protein phosphorylated in Thr231, a residue within the microtubule-binding domain. We observed an evident upregulation of the content of phospho-Tau in the higher Tau bands, including oligomers, that disappeared 24 h after washing out nocodazole (Fig. 1a). Then we analysed the phospho-Tau expression in three PCa cell lines, LNCaP, DU145 and PC3, treated with nocodazole for 18 h and in the time-course following the washout of the cells, according to a typical synchronization protocol (Fig. 1b). All the cell lines demonstrated a similar trend with upregulation of phosphorylated form after 18 h of treatment with nocodazole and a progressive decrease in phospho-Tau during cell cycle re-entry.

### Tau phosphorylation inhibits its binding capacity to β-tubulin

**Fig. 2:**
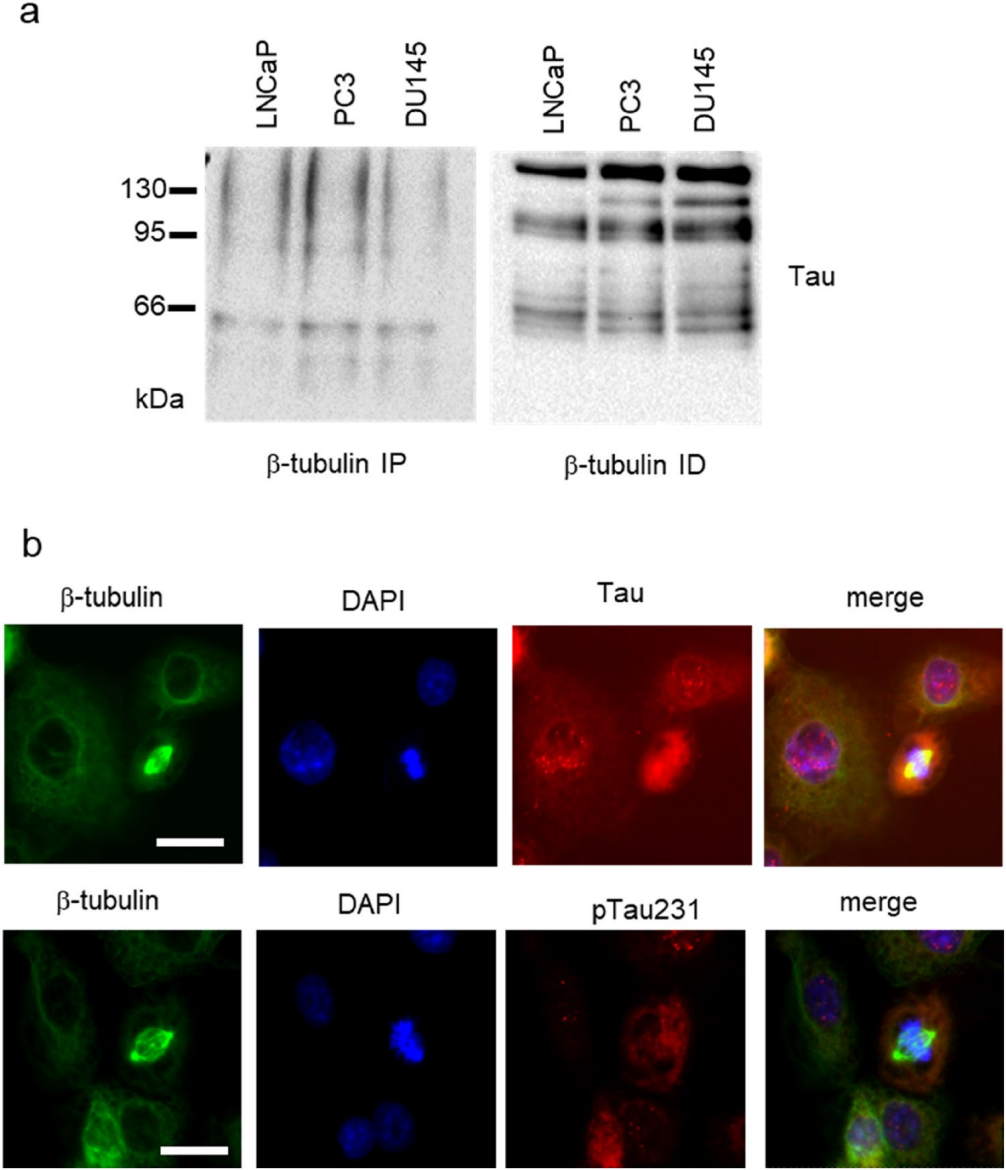
Analysis of the association between β-tubulin and Tau. **a** Total cell lysates from LNCaP, DU145 and PC3 cells were immunoprecipitated for β-tubulin and resulting pellet was probed with anti-Tau antibody (left). The supernatants recovered after immunoprecipitation (immunodepleted samples) were also subjected to western blot for Tau expression (right panel). **b** Representative images from immunofluorescence analysis of DU145 cells stained for the detection of β-tubulin (green), nuclei (blue) total Tau (red, upper row) or Tau phosphorylated at Thr231 (pTau231, red, lower row). The last image of each row is the digital overlay of the previous images (merge). The white bar is a scale of 20 μm

To confirm the binding capacity of tubulin by Tau, we performed the immunoprecipitation of β-tubulin followed by western blot for total Tau in the three PCa cell lines. After immunoprecipitation a main protein form of Tau below 66 KDa was the only detectable band, while higher molecular weight forms including hyper-phosphorylated and oligomeric forms were visible in tubulin-immunodepleted specimens (Fig. 2a). The subcellular localization of Tau by immunofluorescence showed that in interphase cells total protein is visible as a faint diffuse expression in cytoplasm and a spotted expression in the nucleus, while in dividing cells its expression is both overlaying mitotic spindle and diffuse in the cytosol (Fig. 2b, upper panels). On the contrary phospho-Tau was scarcely visible in interphase cells while it was well detectable in dividing cells but not in association with mitotic spindle (Fig. 2b, lower panels).

### Tau phosphorylation status is regulated by cdk1 activity

**Fig. 3:**
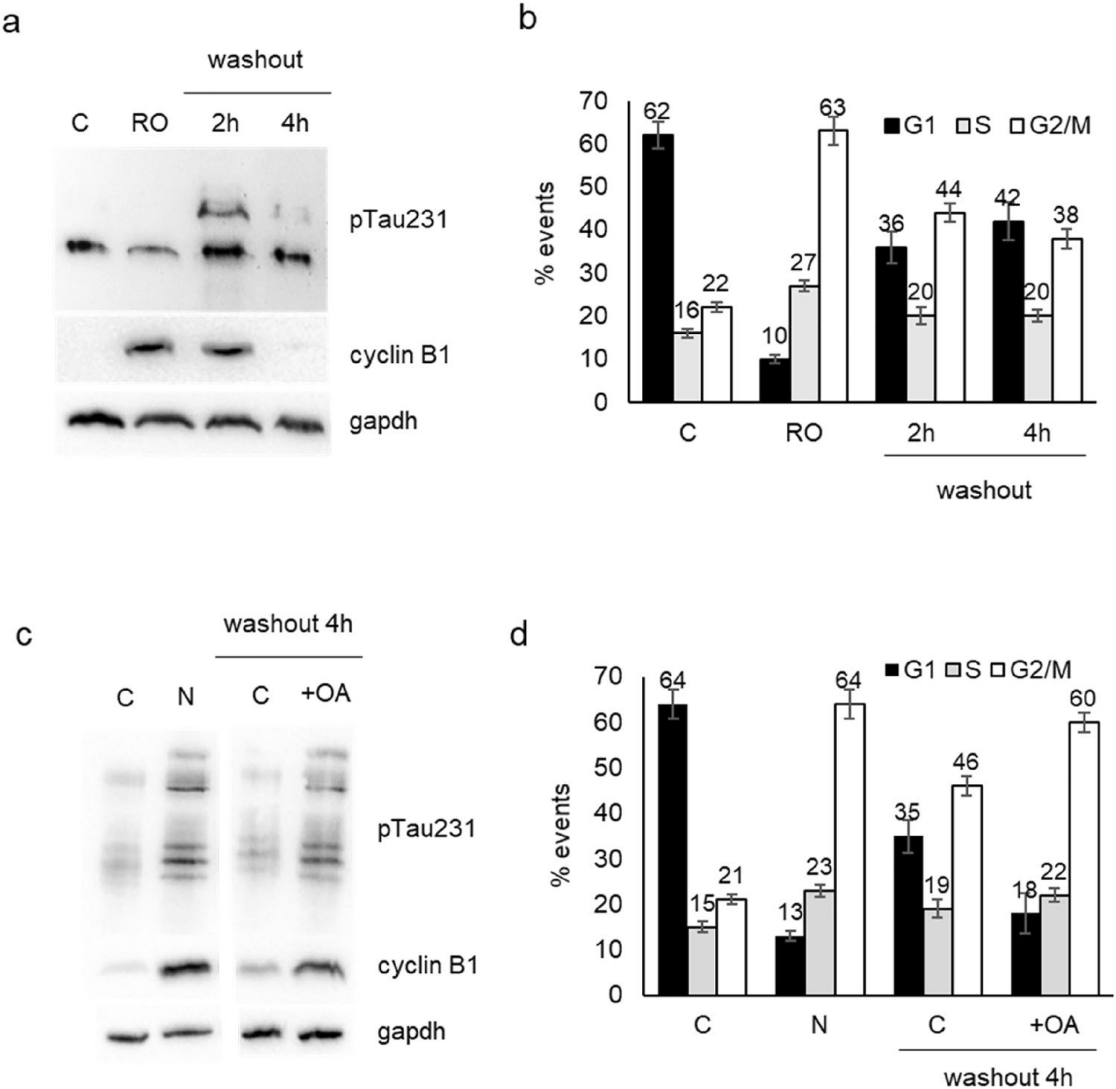
Expression of Tau phosphorylated at Thr231 and cell cycle progression in DU145 cells. **a** Western blot analysis for pTau231 and cyclin B1 in total cell lysates from control cells (C), cells treated for 18 h with RO-3306 (RO) and cells treated for 18 h with RO-3306, washed out and recovered after 2 h or 4 h. Gapdh expression in the same blot was visualized as loading control. **b** DU145 cells subjected to same protocol described in (**a**) were analysed by cytometry after propidium iodide staining and percentage of events in each phase of cell cycle is reported in the histogram. Data represent the mean of three independent experiments ± SD. **c** Western blot analysis for pTau231 and cyclin B1 in total cell lysates from control cells (C), cells treated for 18 h with nocodazole (N) and cells treated for 18 h with nocodazole, washed out, treated (+ OA) or not (C) with okadaic acid and recovered after 4 h. Gapdh expression in the same blot was visualized as loading control. **d** DU145 cells subjected to the same protocol described in (**c**) were analysed by cytometry for cell cycle analysis and percentage of events in each phase is reported in the histogram. Data represent the mean of three independent experiments ± SD

The upregulation of pTau231 in G2/M phase may suggest an indirect regulation by the cell cycle master kinase, cdk1, thus we verified the effect of the cdk1 inhibitor RO-3306 on Tau phosphorylation. We treated DU145 cells with RO-3306 for 18 h and after washout we analysed cells at 2 h and 4 h. The treatment with the inhibitor for 18 h reduced pTau levels respect to untreated cells and cells were blocked in G2/M cell cycle as demonstrated by increased cyclin B1 level (Fig. 3a) and FACS analysis (Fig. 3b). Significantly, in the few hours after washout we assisted at a transitory increase in pTau in parallel with the completion of M phase and re-entry of cells in G1 phase.

In order to further analyse the role of pTau during progression through G2/M phase, we utilized also okadaic acid, an inhibitor of phosphatase PP2a, involved in the dephosphorylation of the residue Thr231. After treatment with nocodazole we performed the washout to facilitate cell cycle re-entry of DU145 cells with or without okadaic acid. Indeed, 4 h after washout we observed that cells treated with okadaic acid expressed higher level of pTau231 and cyclin B1 respect to untreated cells (Fig. 3c). The presence of okadaic acid determined a decreased capacity by cells to re-enter G1 phase after nocodazole washout as demonstrated by cytometry analysis (Fig. 3d).

### Docetaxel induces accumulation of Tau forms phosphorylated at residue Thr231

**Fig. 4:**
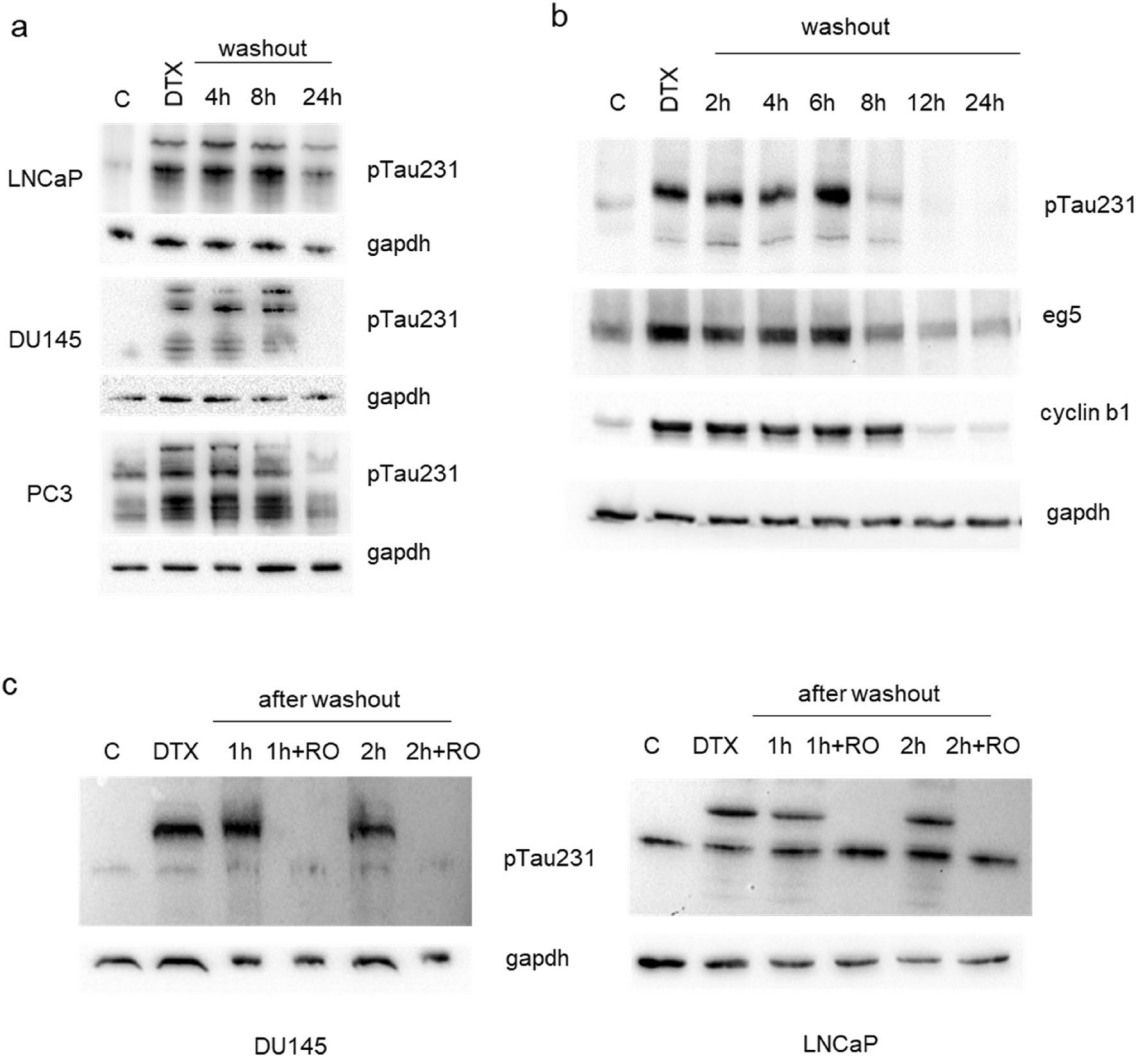
Expression of Tau phosphorylated at Thr231 in PCa cells treated with docetaxel. **a** LNCaP, DU145 and PC3 cells were treated with docetaxel and total cell lysates were recovered at 18 h (DTX) and in a time course after washout (4 h, 8 h, 24 h). For each western blot, gapdh expression was showed as loading control. **b** DU145 cells were treated with docetaxel and total cell lysates were recovered at 18 h (DTX) and in a time course after washout (2 h, 4 h, 6 h, 8 h, 12 h, 24 h). **c** Total cell lysates from DU145 and LNCaP cells were subjected to western blot analysis for the expression pTau231. Cell lines were treated with docetaxel and cell lysates were recovered at 18 h (DTX) and in a time course after washout (1 h and 2 h) in presence or not of RO-3306 (+ RO). For each western blot, gapdh expression was showed as loading control

The treatment of PCa cells with docetaxel determined the upregulation of pTau231, as previously seen with nocodazole, however, the effect was long-lasting respect to nocodazole and a recovery of the basal level of pTau231 was detected only starting 12 h after the washout (Fig. 4a, b). The accumulation of cyclin B1 was accompanied by accumulation also of the eg5 kinesin, a protein essential for spindle assembly, with a restoration of its basal level that paralleled the trend showed by pTau231 and preceded the downmodulation of cyclin B1 (Fig. 4b). Then we aimed at verify whether also in presence of docetaxel the phosphorylation status of Tau was dependent on cdk1 activity. The addition of RO-3306 after washout of docetaxel determined a faster decrease in pTau231 respect to untreated cells confirming that also in presence of docetaxel, Tau phosphorylation was modulated by machinery controlling progression through G2/M (Fig. 4c).

### Cytotoxic effect of docetaxel is counteracted by Tau dephosphorylation

**Fig. 5:**
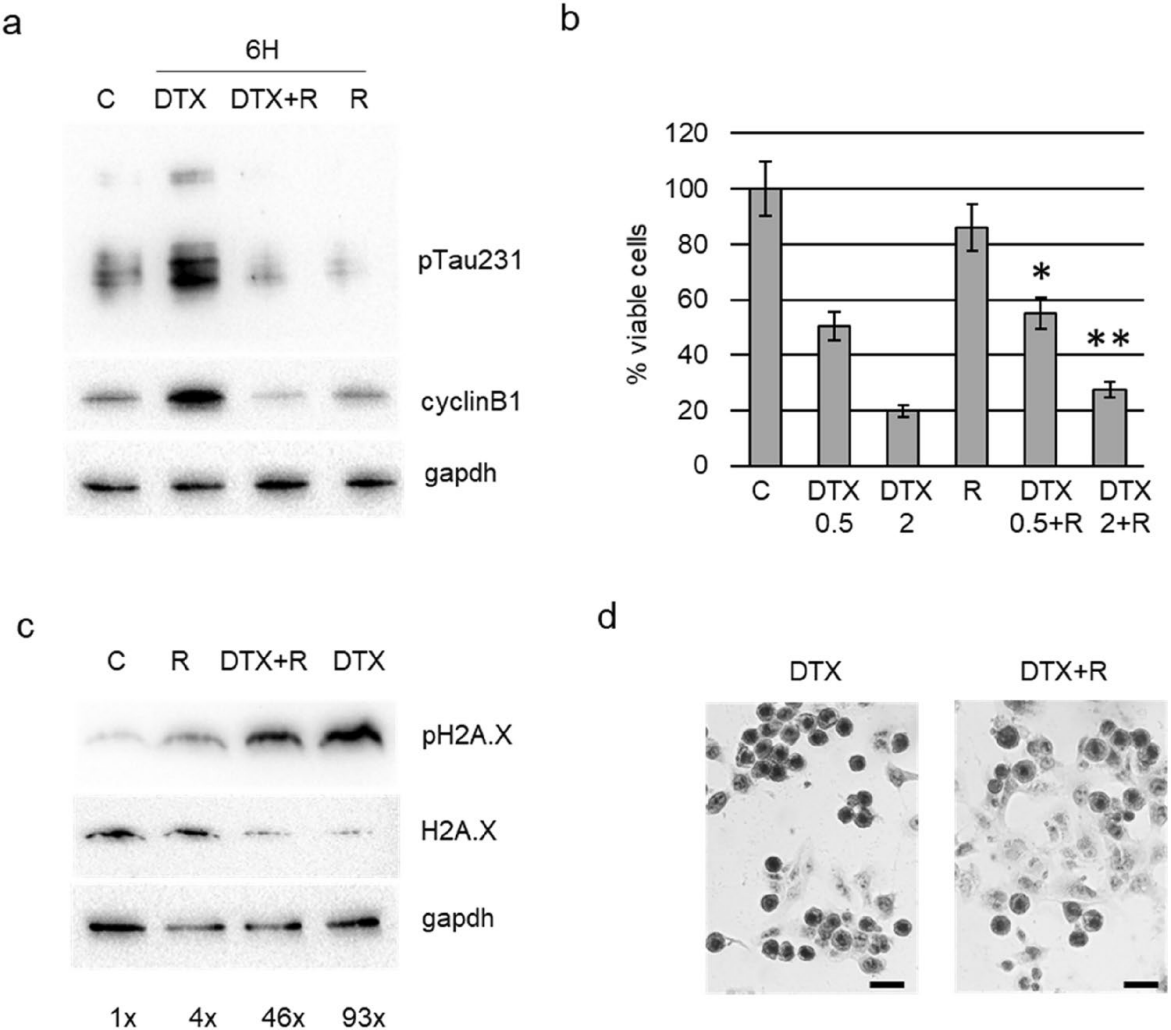
Effect of roscovitine treatment in DU145 cells. **a** Cells were treated for 6 h with docetaxel, with docetaxel plus roscovitine (DTX + R) or with roscovitine (R) and total cell lysates were analysed by western blot for the expression of pTau231, cyclin B1 and gapdh. **b** Viable cells were measured by trypan blue exclusion test after treatment for 24 h with docetaxel 0.5 nM (DTX 0.5), docetaxel 2 nM (DTX 2), roscovitine (R), docetaxel 0.5 nM plus roscovitine (DTX 0.5 + R), docetaxel 2 nM plus roscovitine (DTX 2 + R). Data are represented as percentage considering control (C) as 100%. Results are the mean of three independent experiments ± SD. *p < 0.05 respect DTX 0.5; **p < 0.05 respect DTX 0.5. **c** Cells were treated for 24 h with docetaxel (DTX), with docetaxel plus roscovitine (DTX + R) or with roscovitine (R) and total cell lysates were analysed by western blot for the expression of pH2A.X, H2A.X and gapdh. Densitometric analysis of each pH2A.X band was performed normalizing for its respective H2A.X and gapdh bands and resulting values are showed. **d** Representative images of DU145 cells stained with haematoxylin after treatment for 24 h with 2 nM docetaxel (DTX) or with 2 nM docetaxel plus roscovitine (DTX + R). The black bar is a scale of 20 μm

**Fig. 6:**
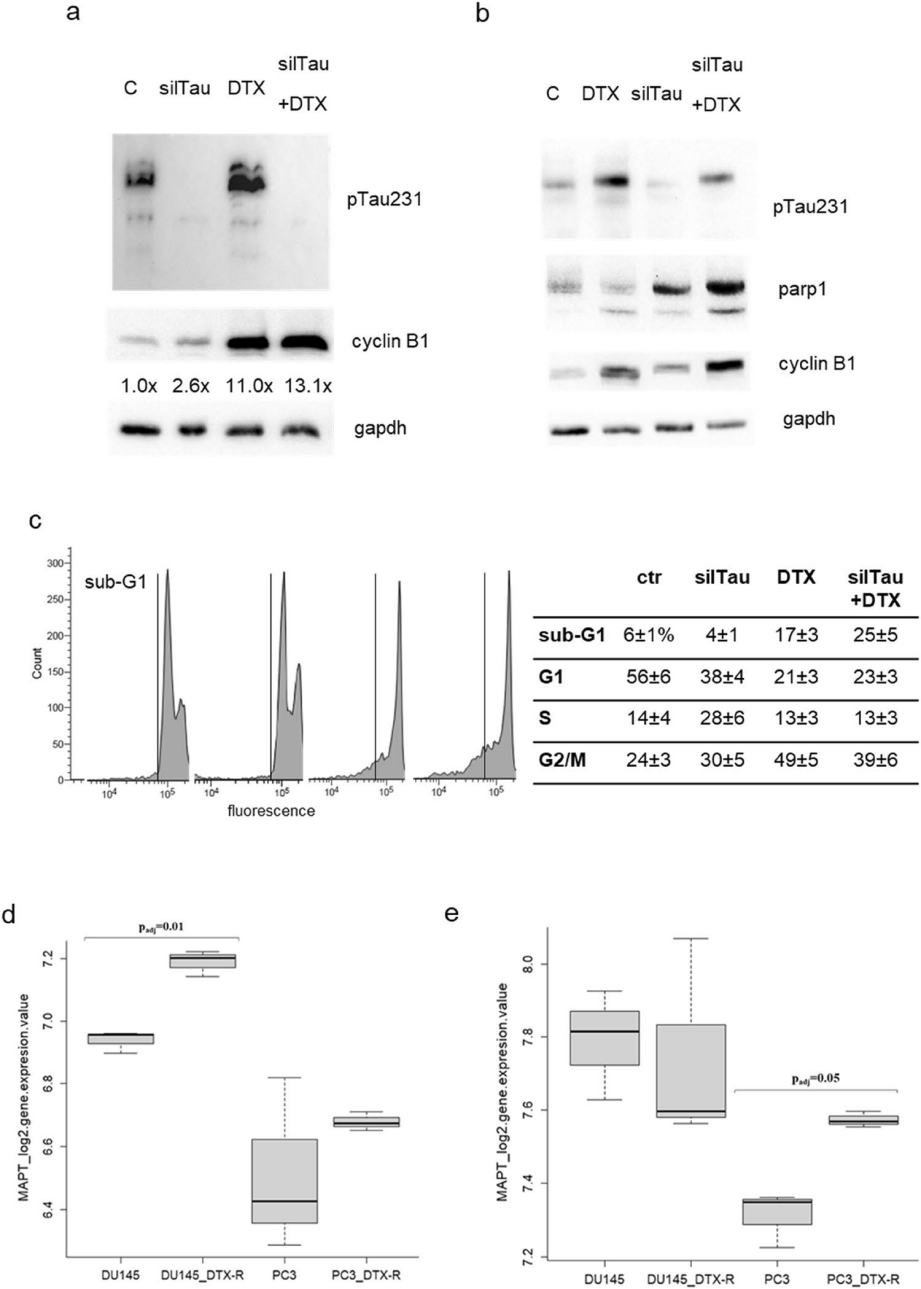
Effect of Tau silencing in DU145 cells. **a** DU145 cells were transfected with siRNA targeting Tau (silTau) or with scrambled siRNA (C), and after 24 h were treated with docetaxel for further 24 h. At the end point cell lysates were analyzed for pTau231, cyclin B1 and gapdh expression. Densitometry of cyclin B1 bands respect to gapdh bands was performed and resulting values are indicated below cyclin B1 blot. **b** DU145 cells were transfected with siRNA targeting Tau (silTau) or with scrambled siRNA (C), and after 24 h were treated with docetaxel for further 24 h. At the end point cell lysates were analyzed for pTau231, parp1, cyclin B1 and gapdh expression. **c** DU145 cells were subjected to the same protocol described in (**a**) and analysed by cytometry after propidium iodide staining. Percentage of events ± SD for each cell cycle phase and in the sub-G1 region are showed in the table, and representative profiles acquired in logarithmic mode are showed in the upper panels, evidencing the sub-G1 region. Data are means of three independent experiments ± SD. **d**, **e** Expression levels, reported as log2 values, of MAPT in DU145 and PC3 prostate cancer cell lines, sensitive (original cell line) and resistant (*R*) to docetaxel (DTX): boxplot showing MAPT expression reported in the GSE33455 dataset (**d**); boxplot showing MAPT expression reported in the GSE158494 dataset (**e**). Statistical significance was calculated by using GEO2R software

Then we aimed at verify the biological consequences of the pharmacological modulation of Tau phosphorylation during the G2/M phase block induced by docetaxel, using roscovitine an inhibitor of cdk5, the kinase responsible for phosphorylation on residue Thr231. Roscovitine was added to DU145 cells that have been pre-treated with docetaxel for 4 h, and then we analysed cells after further 2 h. Roscovitine determined the down-modulation of pTau231 also in presence of docetaxel, and cells treated with combination expressed lower level of cyclin B1 respect to cells treated only with docetaxel (Fig. 5a). When we analysed the effect on cell viability of the same treatment protocol after 24 h, we observed a small but significant increase in the number of viable cells in the combination treatment respect to docetaxel alone (Fig. 5b). To verify the genotoxic effect of the combination treatment, we analysed the expression of double strand break marker, pH2A.X, in relation with the total amount of H2A.X. Twenty-four hours of docetaxel treatment induced a marked upregulation of pH2A.X in DU145 cells respect to both control cells and cells treated with roscovitine (Fig. 5c). The addition of roscovitine to docetaxel was able to counteract the upregulation of pH2A.X, decreasing of about 50% its expression levels. When observed after haematoxylin staining by microscopy, a higher number of well adhered cells was visible in presence of roscovitine respect to experimental point treated only with docetaxel (Fig. 5d). Significantly, aberrant nuclei were frequent in these cells. Then we verified the effect of knockdown of Tau protein by transient silencing. DU145 cells incubated for 48 h with Tau-targeting siRNA or with random RNA sequences were treated with docetaxel for further 24 h. Western blot analysis confirmed the absence of pTau and showed the upregulation of cyclin B1 in silenced cells respect to respective controls both in untreated and docetaxel-treated cells (Fig. 6a). The protective role of Tau was demonstrated by the cleavage of parp1, a key responder downstream of DNA damage, visible mainly in silenced cells treated with docetaxel (Fig. 6b). The analysis of cell cycle by cytometry confirmed the presence of a higher number of events in G2/M in silenced cells respect to respective controls and in addition revealed a significant increase in subG1 events in silenced cells treated with docetaxel respect to control cells treated with docetaxel (Fig. 6c). Gene Expression Omnibus (GEO) datasets were used to evaluate the expression levels of MAPT in DU145, PC3 and 22Rv1 cell lines sensitive and resistant to docetaxel. As shown in Fig. 6d, e, upregulation of median MAPT expression was found in docetaxel resistant DU145 and PC3 cells compared to original cell line in the GSE33455 dataset, but a statistically significant upregulation was seen only in DU145 resistant cells (padj = 0.01) (Fig. 6d). Upregulation of MAPT was confirmed in the GSE158494 dataset for PC3 resistant cells compared to original cell line (padj = 0.05) (Fig. 6e). No statistically significant differences were observed between docetaxel sensitive and resistant DU145 and 22Rv1 cell lines in the GSE36135 dataset (data not shown).

## Discussion

Our current knowledge about Tau biology is largely based upon results obtained from neuronal models with particular interest for neurodegenerative diseases. These studies have demonstrated the central role of Tau in maintaining cytoarchitecture and intracellular transport in neurons. However, it is significant that also in post mitotic models, as neurons are, aberrant expression of Tau has been linked to cell cycle anomalies. Indeed, it is well known that in the brains of Alzheimer’s disease (AD) patients, vulnerable neurons re-enter the cell cycle and attempt to progress through it with aberrant consequences, such as abortive cell death (Bonda et al. 2010). Although, the mechanism underlying deregulation of cell cycle in these experimental models surely involves the activation of different molecular pathways, the existence of a solid and bi-univocal correlation between Tau and cell cycle in AD has been suggested. On one hand cell cycle related proteins have been shown to directly phosphorylate Tau (Baumann et al. 1993). Conversely, forced expression of non-mutant human Tau in mouse model was associated with the aberrant appearance of cell-cycle and the initiation of DNA synthesis (Andorfer et al. 2005). These evidence have prompted some authors to suggest that neurodegenerative diseases could resemble cancer in their early aetiology, involving a loss of cell-cycle control.

What seems mostly relevant in explaining the pathologic role of Tau in modulation of cell cycle progression is the ratio of the various post-translational forms of protein. Because Tau is differentially phosphorylated during the cell cycle it is plausible to hypothesize the presence of a control mechanism involving kinases and phosphatases normally associated with cell cycle checkpoints (Pope et al. 1994). Tau has a low level of phosphorylation in interphase or in G0 phase when it is mainly bound to microtubules, while, at the onset of DNA duplication, an increase in phosphorylation correlates to a reduction in its affinity for microtubules (Preuss et al. 1995). Tau contains 85 potential phosphorylation sites, with different amino acids that were described as critically important for the interaction with microtubules. Indeed, during mitosis, a substantial fraction of Tau is phosphorylated at residues Thr231 Ser235 and Ser262, and these phosphorylated forms are found free in the cytoplasm (Alquezar et al. 2020). Our attention was focused on the residue Thr231 because its phosphorylation not only regulates microtubule binding but is also important for the pathologic role of Tau (Alonso et al. 2010). Thr231 is the main target of cdk5-p25, that is considered the pathologic form of the kinase (Paudel et al. 1993). However, contrary to other cdks that are activated at a particular cell cycle phase, cdk5 is expressed in many tissues in a constitutive manner. Interestingly, cdk5 was demonstrated to be necessary for the control of neuronal cell cycle arrest and differentiation (Cicero and Herrup 2005). In PCa cells, cdk5 promotes cell growth in an androgen receptor- independent way and stimulate cancer progression (Lindqvist et al. 2015). In our study, we demonstrated that roscovitine, an inhibitor of cdk5, can significantly suppress the phosphorylation at residue Thr231 and this effect is evident both in control cells and in cells blocked in G2/M by docetaxel. While this result confirms a critical role of cdk5 in controlling Tau function, the biological consequences of the combination treatment docetaxel plus roscovitine were unexpected. The addition of roscovitine to cells blocked by docetaxel protected partially them from the cytotoxic effect of the taxane and in particular seems to sustain DNA integrity and exit from mitosis. This result suggests that phosphorylation of Tau is fundamental in maintaining cells in G2/M phase and the existence of a cell-cycle dependent mechanism controlling phosphorylation status of Tau.

Our data indicate that a central role in modulating Tau phosphorylation in G2/M is exerted by the master kinase cdk1. Indeed, the inhibition of the kinase activity of cdk1 by RO-3306 determined the arrest of cells in G2/M phase in presence of non-phosphorylated Tau. The effect on Tau phosphorylation by inhibition of cdk1 was evident also in cells that were previous blocked by nocodazole. Interestingly the progression through cell cycle after washout of the cdk1 inhibitor was associated with a transient increased pTau231 expression suggesting the importance of Tau phosphorylation for the correct completion of the M phase. Although current evidence does not support a direct role for cdk1 in phosphorylating Tau, we may hypothesize the presence of an indirect mechanism. It is well known that the realization of mitosis is the result of a fine regulation at the kinases and phosphatases level. In particular, the phosphatase PP2A has emerged as cdk1 counteracting enzyme in different animal cell models. In fact, it is supposed that PP2A and cdk1 function as reciprocal guide during entry and exit from the mitosis (Bollen et al. 2009; Yin et al. 2019). Interestingly the same authors demonstrated that the deletion of SET nuclear proto-oncogene (2PP2A), a PP2A inhibitor, resulted in aberrant spindle formation and chromosome mis-segregation during mitosis. In addition, available data support a critical role for the SET-PP2A signalling axis also in PCa progression (Hu et al. 2015). In our study we verified that the addition of okadaic acid, a PP2A inhibitor, after synchronization of cell by nocodazole counteracted the capacity of cells to complete mitosis. The dependence of Tau phosphorylation on cdk1 activity and PP2A inhibition suggests that during G2 and M phase, at least until pro-metaphase, Tau is maintained prevalently in the phosphorylated form, detached from microtubules. An interesting evidence in agreement with this hypothesis derives from in situ analysis of chromosomal pattern in AD tissue demonstrating that in AD neurons S phase is nearly accomplished, but mitosis is not initiated (Yang et al. 2001). The entry in metaphase in parallel with the inhibition of cdk1 activity and reactivation of PP2A, may determine the up-regulation of the dephosphorylated forms of Tau, favouring its tubulin-binding activity, and its role as guide for spindle formation. This hypothesis could explain why events determining the absence of dephosphorylated Tau, such as Tau knockdown, or okadaic acid treatment are associated with difficulties in progression through mitosis.

Mutational studies have clearly demonstrated that total or even partial loss of Tau function determined aneuploidy in mouse and human neuronal models (Caneus et al. 2018). A possible explanation for this effect derived from the evidence that loss of Tau proteostasis can determine mitotic spindle abnormalities. Indeed, Tau, beside its role in microtubule stabilization, can interact and modulate the activity of the mitotic motor kinesin Eg5 (Bougé and Parmentier 2016). In our previous study we have observed spindle abnormalities in consequence of the accumulation of oligomeric forms of Tau and we have demonstrated that elimination of these forms by autophagy is important in maintaining proliferative capacity in PCa cells (Martellucci et al. 2021). In this work we verified a positive correlation between the accumulation of Eg5 and pTau, and this event was evident in cells blocked in G2/M phase by docetaxel treatment, confirming that in these cells the perturbation of mitotic spindle assembly involves also Eg5 homeostasis. Although there is relatively little information to explain the toxic role of soluble Tau oligomers, it is widely accepted that their accumulation represents an early pathological marker. The formation of Tau oligomers is closely linked to Tau phosphorylation status, and when regulation of the balance between phosphorylation and de-phosphorylation is lost, leading to abnormal hyper-phosphorylation, the resulting effect is the dissociation from microtubule and the beginning of the cascade leading to accumulation of progressively more complex oligomeric forms.

Although numerous data have accumulated about the upregulation or de novo expression of Tau protein in different cancer tissues, efforts in individuating its prognostic role have been disappointed. Tau expression in cancers has been associated to resistance to taxanes. In agreement with the suggested role of Tau in modulating the formation of mitotic spindle the potential antagonistic activity against tubulin-directed antimitotic drug is not surprising. Indeed, centrosome clustering and activity of motor kinesins has been proposed as one of the possible mechanism of resistance to docetaxel in solid tumours (Rath and Kozielski 2012; Wiltshire et al. 2010). In clinical settings, Tau upregulation was repetitively individuated in molecular signatures from cancers resistant to taxanes (Smoter et al. 2011). However, Tau expression analyses obtained from different non-neurological tumours, including breast, ovarian, colorectal and prostate generated contradictory results, and large clinical studies have confuted early evidences about diagnostic or predictive value of Tau protein expression (Pentheroudakis et al. 2009; Irshad et al. 2014). Our analysis of available mRNA expression datasets in PCa cell lines demonstrated a trend in the upregulation of Tau in docetaxel resistant cells respect to original cells, but not in all cases, suggesting that Tau upregulation could participate to create a resistant phenotype only in specific circumstances. In our opinion, a definitive conclusion about the predictive role of Tau could be obtained only with a more robust knowledge about the mechanisms underlying its post-translational modulation during mitosis. In fact, to date, preclinical results have proposed very dissimilar biological bases for a potential association of Tau expression with taxane resistance. A first hypothesis concerns the similarity in the sequence allowing the binding to tubulin between Tau and taxol. This aspect has induced researchers to hypothesize a competitive mechanism in assembly of microtubules, with Tau counteracting the stabilizing activity of taxol (Kar et al. 2003). In addition, Tau has been implicated also in maintenance of the DNA integrity and nuclear structure suggesting a protective role of Tau from DNA damage induced by taxanes (Asada-Utsugi et al. 2022). Besides, Tau has been proposed to interact with key signalling pathways involved in cancer resistance to therapy. Indeed, in neuroblastoma model with depleted Tau, a reduced capacity to stabilize p53 after DNA damage was observed (Sola et al. 2020). In addition Tau binds to PI3K (phosphatidylinositol 3-kinase) and it has been shown to activate MAPK (Mitogen-activated protein kinase) in response to NGF (nerve growth factor) and EGF (epidermal growth factor) (Souter and Lee 2009; Leugers and Lee 2010).

With our study we contributed to elucidate the regulation of Tau phosphorylation during cell cycle in a cancer cell model, showing a fine-tuned regulation that is important for the correct realization of the mitosis. On this background, we suggest the oncogenic relevance of those mechanisms involving kinases and phosphatases that control Tau phosphorylation rather than the mere expression of the protein. As suggested by our data, the post-translational control of Tau could be important also in explaining sensitivity to taxanes, with dephosphorylated Tau that protects cancer cells in the short term from docetaxel-induced cytotoxicity.

## Data Availability

Most data generated or analyzed during this study are included in this article. The datasets and materials used and/or analyzed during the current study are available from the corresponding author on reasonable request.
